# Supplementary material for: DNA metabarcoding reveals diet diversity and niche partitioning by two sympatric herbivores in summer
Source: PeerJ. 2024 Dec 23;12:e18665. doi: 10.7717/peerj.18665 (PMC11670756; doi:10.7717/peerj.18665)
Supplement: Supplemental Information 1 [file peerj-12-18665-s001.docx]

**Supplementary materials Table S1** Alpha diversity indices including Observed_ASVs, Chao1, Shannon, Faith_pd, Simpson, and Pielou of sika deer sample group

| **Sample** | **Observed_ASVs** | **Chao1** | **Shannon** | **Faith_pd** | **Simpson** | **Pielou** |
| --- | --- | --- | --- | --- | --- | --- |
| Sika deer_01 | 134 | 147.15 | 3.40 | 4.94 | 0.81 | 0.62 |
| Sika deer_02 | 56 | 66.11 | 2.01 | 3.96 | 0.57 | 0.36 |
| Sika deer_03 | 143 | 176.30 | 2.93 | 5.63 | 0.66 | 0.53 |
| Sika deer_04 | 159 | 194.43 | 4.49 | 6.82 | 0.93 | 0.82 |
| Sika deer_05 | 37 | 38.88 | 1.28 | 2.82 | 0.36 | 0.23 |
| Sika deer_07 | 151 | 176.83 | 3.99 | 5.46 | 0.87 | 0.73 |
| Sika deer_08 | 144 | 166.24 | 3.84 | 5.96 | 0.84 | 0.70 |
| Sika deer_09 | 37 | 41.00 | 1.87 | 3.11 | 0.51 | 0.34 |
| Sika deer_10 | 67 | 101.00 | 1.69 | 4.47 | 0.50 | 0.30 |
| Sika deer_11 | 122 | 129.92 | 2.87 | 5.54 | 0.67 | 0.52 |
| Sika deer_12 | 154 | 191.06 | 4.20 | 6.22 | 0.91 | 0.76 |
| Sika deer_13 | 49 | 58.00 | 2.12 | 2.99 | 0.68 | 0.38 |
| Sika deer_14 | 77 | 105.88 | 3.34 | 3.88 | 0.84 | 0.61 |
| Sika deer_15 | 68 | 93.50 | 2.09 | 3.92 | 0.57 | 0.388 |
| Sika deer_16 | 44 | 51.00 | 1.20 | 2.95 | 0.34 | 0.21 |
| Sika deer_17 | 39 | 45.00 | 1.44 | 2.22 | 0.40 | 0.26 |
| Sika deer_18 | 133 | 176.15 | 4.06 | 5.96 | 0.90 | 0.74 |
| Sika deer_19 | 147 | 198.25 | 4.12 | 5.85 | 0.88 | 0.75 |
| Sika deer_20 | 35 | 37.33 | 1.11 | 2.57 | 0.37 | 0.20 |
| Sika deer_21 | 68 | 89.11 | 2.63 | 4.19 | 0.74 | 0.48 |
| Sika deer_22 | 164 | 187.25 | 4.25 | 6.64 | 0.91 | 0.77 |
| Sika deer_23 | 45 | 52.00 | 1.17 | 3.19 | 0.30 | 0.21 |
| Sika deer_24 | 146 | 181.77 | 4.07 | 6.05 | 0.89 | 0.74 |
| Sika deer_25 | 45 | 52.00 | 1.07 | 3.27 | 0.27 | 0.19 |
| Sika deer_26 | 157 | 183.25 | 4.31 | 6.94 | 0.92 | 0.78 |
| Sika deer_27 | 105 | 122.27 | 3.08 | 5.55 | 0.78 | 0.56 |
| Sika deer_29 | 100 | 111.18 | 2.35 | 5.14 | 0.68 | 0.43 |
| Sika deer_30 | 159 | 231.77 | 3.94 | 6.28 | 0.87 | 0.72 |
| **Mean±SE** | **99.46±9.19** | **121.59±11.63** | **2.81±0.22** | **4.73±0.27** | **0.67±0.04** | **0.51±0.04** |
