# Supplementary material for: DNA metabarcoding reveals diet diversity and niche partitioning by two sympatric herbivores in summer
Source: PeerJ. 2024 Dec 23;12:e18665. doi: 10.7717/peerj.18665 (PMC11670756; doi:10.7717/peerj.18665)
Supplement: Supplemental Information 2 [file peerj-12-18665-s002.docx]

| **Sample** | **Observed_otus** | **Chao1** | **Shannon** | **Faith_pd** | **Simpson** | **Pielou** |
| --- | --- | --- | --- | --- | --- | --- |
| Reeves' Muntjac_01 | 78 | 90.00 | 3.18 | 3.39 | 0.82 | 0.59 |
| Reeves' Muntjac_02 | 70 | 81.00 | 3.03 | 3.54 | 0.80 | 0.56 |
| Reeves' Muntjac_03 | 110 | 127.50 | 3.06 | 4.48 | 0.78 | 0.56 |
| Reeves' Muntjac_04 | 98 | 113.00 | 2.74 | 4.66 | 0.73 | 0.50 |
| Reeves' Muntjac_05 | 20 | 20.60 | 1.03 | 1.29 | 0.30 | 0.19 |
| Reeves' Muntjac_06 | 22 | 32.00 | 0.46 | 2.50 | 0.14 | 0.08 |
| Reeves' Muntjac_07 | 111 | 119.27 | 3.19 | 4.49 | 0.69 | 0.59 |
| Reeves' Muntjac_08 | 64 | 84.00 | 0.76 | 3.89 | 0.16 | 0.14 |
| Reeves' Muntjac_09 | 47 | 58.25 | 3.19 | 2.01 | 0.86 | 0.59 |
| Reeves' Muntjac_10 | 41 | 46.25 | 1.82 | 2.50 | 0.54 | 0.33 |
| Reeves' Muntjac_11 | 25 | 39.00 | 2.34 | 1.63 | 0.76 | 0.43 |
| Reeves' Muntjac_12 | 30 | 37.20 | 0.50 | 2.80 | 0.13 | 0.09 |
| Reeves' Muntjac_13 | 62 | 69.86 | 3.32 | 3.53 | 0.86 | 0.61 |
| Reeves' Muntjac_14 | 59 | 75.50 | 2.58 | 3.67 | 0.77 | 0.47 |
| Reeves' Muntjac_15 | 107 | 137.00 | 2.40 | 5.34 | 0.60 | 0.44 |
| Reeves' Muntjac_16 | 69 | 96.20 | 2.75 | 3.90 | 0.78 | 0.51 |
| Reeves' Muntjac_17 | 100 | 117.65 | 2.64 | 5.03 | 0.71 | 0.49 |
| Reeves' Muntjac_18 | 83 | 100.50 | 2.95 | 4.15 | 0.74 | 0.54 |
| Reeves' Muntjac_20 | 37 | 39.50 | 2.79 | 3.02 | 0.78 | 0.51 |
| Reeves' Muntjac_21 | 148 | 183.43 | 4.36 | 5.37 | 0.91 | 0.80 |
| Reeves' Muntjac_22 | 94 | 124.00 | 1.50 | 4.85 | 0.35 | 0.27 |
| Reeves' Muntjac_23 | 32 | 37.25 | 1.44 | 2.73 | 0.43 | 0.26 |
| Reeves' Muntjac_24 | 77 | 87.91 | 2.62 | 4.61 | 0.69 | 0.48 |
| Reeves' Muntjac_25 | 100 | 130.00 | 2.04 | 5.35 | 0.50 | 0.37 |
| Reeves' Muntjac_26 | 71 | 82.67 | 1.15 | 4.40 | 0.25 | 0.21 |
| Reeves' Muntjac_27 | 21 | 21.50 | 1.14 | 1.92 | 0.31 | 0.21 |
| Reeves' Muntjac_28 | 118 | 158.63 | 3.68 | 4.55 | 0.86 | 0.68 |
| Reeves' Muntjac_29 | 100 | 128.88 | 4.02 | 5.25 | 0.90 | 0.74 |
| **Mean±SE** | **71.21±6.54** | **87.09±8.19** | **2.38±0.20** | **3.74±0.23** | **0.61±0.05** | **0.44±0.04** |

**Supplementary materials Table S2** Alpha diversity indices including Observed_ASVs, Chao1, Shannon, Faith_pd, Simpson, and Pielou of Reeves’ muntjac sample group
