# Supplementary material for: DNA metabarcoding reveals diet diversity and niche partitioning by two sympatric herbivores in summer
Source: PeerJ. 2024 Dec 23;12:e18665. doi: 10.7717/peerj.18665 (PMC11670756; doi:10.7717/peerj.18665)
Supplement: Supplemental Information 3 [file peerj-12-18665-s003.docx]

**Supplementary materials Table S3** Common ASVs between sika deer and Reeves’ muntjac with top 100 ASVs in abundance

| Number | Species | Order | Family | Genus | Abundance |
| --- | --- | --- | --- | --- | --- |
| 1 | *Smilax china* | Liliales | Smilacaceae | *Smilax* | 195491 |
| 2 | *Rubus* spp. | Rosales | Rosaceae | *Rubus* | 141633 |
| 3 | *Dicranum scoparium* | Dicranales | Dicranaceae | *Dicranum* | 112611 |
| 4 | *Loropetalum chinense* | Saxifragales | Hamamelidaceae | *Loropetalum* | 76938 |
| 5 | *Sassafras tzumu* | Laurales | Lauraceae | *Sassafras* | 60571 |
| 6 | *Phyllostachys edulis* | Poales | Poaceae | *Phyllostachys* | 46947 |
| 7 | *Cunninghamia lanceolata* | Cupressales | Cupressaceae | *Cunninghamia* | 37246 |
| 8 | *Pohlia elongata* | Bryales | Mniaceae | *Pohlia* | 37059 |
| 9 | *Alangium chinense* | Cornales | Cornaceae | *Alangium* | 28192 |
| 10 | *Rumex acetosa* | Caryophyllales | Polygonaceae | *Rumex* | 28114 |
| 11 | *Rhododendron simsii* | Ericales | Ericaceae | *Rhododendron* | 26618 |
| 12 | *Rhus chinensis* | Sapindales | Anacardiaceae | *Rhus* | 24792 |
| 13 | *Erigeron annuus* | Asterales | Asteraceae | *Erigeron* | 24710 |
| 14 | *Premna microphylla* | Lamiales | Lamiaceae | *Premna* | 24591 |
| 15 | *Rosa laevigata* | Rosales | Rosaceae | *Rosa* | 23354 |
| 16 | *Persicaria perfoliata* | Caryophyllales | Polygonaceae | *Persicaria* | 21179 |
| 17 | *Wisteria sinensis* | Fabales | Fabaceae | *Wisteria* | 21087 |
| 18 | *Glyphomitrium* sp. | Isobryales | Glyphomitriaceae | *Glyphomitrium* | 19243 |
| 19 | *Rubus coreanus* | Rosales | Rosaceae | *Rubus* | 19127 |
| 20 | *Setaria viridis* | Poales | Poaceae | *Setaria* | 18328 |
| 21 | *Persicaria maculosa* | Caryophyllales | Polygonaceae | *Persicaria* | 16967 |
| 22 | *Digitaria sanguinalis* | Poales | Poaceae | *Digitaria* | 15649 |
| 23 | *Lespedeza bicolor* | Fabales | Fabaceae | *Lespedeza* | 15056 |
| 24 | *Quercus fabri* | Fagales | Fagaceae | *Quercus* | 14838 |
| 25 | *Bidens pilosa* | Asterales | Asteraceae | *Bidens* | 14244 |
| 26 | *Schima superba* | Ericales | Theaceae | *Schima* | 12475 |
| 27 | *Broussonetia papyrifera* | Rosales | Moraceae | *Broussonetia* | 12403 |
| 28 | *Carpesium abrotanoides* | Asterales | Asteraceae | *Carpesium* | 11892 |
| 29 | *Platycarya strobilacea* | Fagales | Juglandaceae | *Platycarya* | 11605 |
| 30 | *Oxalis corniculata* | Oxalidales | Oxalidaceae | *Oxalis* | 11383 |
| 31 | *Ligustrum quihoui* | Lamiales | Oleaceae | *Ligustrum* | 10239 |
| 32 | *Zea mays* | Poales | Poaceae | *Zea* | 9973 |
| 33 | *Prunus mume* | Rosales | Rosaceae | *Prunus* | 9272 |
| 34 | *Diospyros kaki* | Ericales | Ebenaceae | *Diospyros* | 7770 |
| 35 | *Lophatherum gracile* | Poales | Poaceae | *Lophatherum* | 6679 |
| 36 | *Vaccinium bracteatum* | Ericales | Ericaceae | *Vaccinium* | 6226 |
| 37 | *Phyllanthus urinaria* | Malpighiales | Phyllanthaceae | *Phyllanthus* | 6012 |
| 38 | *Ficus erecta* | Rosales | Moraceae | *Ficus* | 5513 |
| 39 | *Malus pumila* | Rosales | Rosaceae | *Malus* | 4531 |
| 40 | *Abelia chinensis* | Dipsacales | Caprifoliaceae | *Abelia* | 4386 |
| 41 | *Spiraea chinensis* | Rosales | Rosaceae | *Spiraea* | 4376 |
| 42 | *Triadica sebifera* | Malpighiales | Euphorbiaceae | *Triadica* | 6961 |
| 43 | *Miscanthus floridulus* | Poales | Poaceae | *Miscanthus* | 3975 |
| 44 | *Dalbergia assamica* | Fabales | Fabaceae | *Dalbergia* | 3925 |
| 45 | *Maclura tricuspidata* | Rosales | Moraceae | *Maclura* | 3616 |
| 46 | *Lactuca indica* | Asterales | Asteraceae | *Lactuca* | 3237 |
| 47 | *Euscaphis japonica* | Crossosomatales | Staphyleaceae | *Euscaphis* | 3167 |
| 48 | *Isodon nervosus* | Lamiales | Lamiaceae | *Isodon* | 2438 |
| 49 | *Melia azedarach* | Sapindales | Meliaceae | *Melia* | 2230 |
| 50 | *Vitis sinocinerea* | Vitales | Vitaceae | *Vitis* | 2119 |
| 51 | *Pueraria montana* | Fabales | Fabaceae | *Pueraria* | 1830 |
| 52 | *Deyeuxia pyramidalis* | Poales | Poaceae | *Deyeuxia* | 1644 |
| 53 | *Paulownia catalpifolia* | Lamiales | Paulowniaceae | *Paulownia* | 1632 |
| 54 | *Galium aparine* | Gentianales | Rubiaceae | *Galium* | 1491 |
| 55 | *Sabia japonica* | Proteales | Sabiaceae | *Sabia* | 1418 |
| 56 | *Triticum aestivum* | Poales | Poaceae | *Triticum* | 1406 |
| 57 | *Elaeagnus umbellata* | Rosales | Elaeagnaceae | *Elaeagnus* | 1316 |
| 58 | *Callicarpa bodinieri* | Lamiales | Lamiaceae | *Callicarpa* | 1197 |
| 59 | *Pistacia chinensis* | Sapindales | Anacardiaceae | *Pistacia* | 1178 |
| 60 | *Sloanea sinensis* | Oxalidales | Elaeocarpaceae | *Sloanea* | 1130 |
| 61 | *Viola inconspicua* | Malpighiales | Violaceae | *Viola* | 1007 |
| 62 | *Debregeasia orientalis* | Rosales | Urticaceae | *Debregeasia* | 921 |
| 63 | *Euonymus fortunei* | Celastrales | Celastraceae | *Euonymus* | 852 |
| 64 | *Itea chinensis* | Saxifragales | Iteaceae | *Itea* | 847 |
| 65 | *Scleromitrion diffusum* | Gentianales | Rubiaceae | *Scleromitrion* | 804 |
| 66 | *Grewia biloba* | Malvales | Malvaceae | *Grewia* | 794 |
| 67 | *Poa pratensis* | Poales | Poaceae | *Poa* | 750 |
| 68 | *Mallotus barbatus* | Malpighiales | Euphorbiaceae | *Mallotus* | 747 |
| 69 | *Eleutherococcus nodiflorus* | Apiales | Araliaceae | *Eleutherococcus* | 715 |
| 70 | *Deutzia* sp. | Cornales | Hydrangeaceae | *Deutzia* | 652 |
| 71 | *Ipomoea triloba* | Solanales | Convolvulaceae | *Ipomoea* | 635 |
| 72 | *Veronica persica* | Lamiales | Plantaginaceae | *Veronica* | 593 |
| 73 | *Albizia kalkora* | Fabales | Fabaceae | *Albizia* | 567 |
| 74 | *Arundinella hirta* | Poales | Poaceae | *Arundinella* | 562 |
| 75 | *Lysimachia clethroides* | Ericales | Primulaceae | *Lysimachia* | 537 |
| 76 | *Indigofera tinctoria* | Fabales | Fabaceae | *Indigofera* | 515 |
| 77 | *Eurya nitida* | Ericales | Pentaphylacaceae | *Eurya* | 514 |
| 78 | *Celtis biondii* | Rosales | Cannabaceae | *Celtis* | 496 |
| 79 | *Camphora officinarum* | Laurales | Lauraceae | *Camphora* | 487 |
| 80 | *Pleuropterus multiflorus* | Caryophyllales | Polygonaceae | *Pleuropterus* | 454 |
| 81 | *Lygodium japonicum* | Schizaeales | Lygodiaceae | *Lygodium* | 412 |
| 82 | *Justicia procumbens* | Lamiales | Acanthaceae | *Justicia* | 409 |
| 83 | *Fortunearia sinensis* | Saxifragales | Hamamelidaceae | *Fortunearia* | 389 |
| 84 | *Corylopsis sinensis* | Saxifragales | Hamamelidaceae | *Corylopsis* | 387 |
| 85 | *Stephania tetrandra* | Ranunculales | Menispermaceae | *Stephania* | 347 |
| 86 | *Cercidiphyllum japonicum* | Saxifragales | Cercidiphyllaceae | *Cercidiphyllum* | 314 |
| 87 | *Panicum bisulcatum* | Poales | Poaceae | *Panicum* | 283 |
| 88 | *Styrax japonicus* | Ericales | Styracaceae | *Styrax* | 275 |
| 89 | *Aphananthe aspera* | Rosales | Cannabaceae | *Aphananthe* | 265 |
| 90 | *Prunus* sp. | Rosales | Rosaceae | *Prunus* | 261 |
| 91 | *Paraprenanthes sororia* | Asterales | Asteraceae | *Paraprenanthes* | 259 |
| 92 | *Broussonetia kaempferi* | Rosales | Moraceae | *Broussonetia* | 242 |
| 93 | *Trema* sp. | Rosales | Cannabaceae | *Trema* | 231 |
| 94 | *Tetradium ruticarpum* | Sapindales | Rutaceae | *Tetradium* | 226 |
| 95 | *Viburnum dilatatum* | Dipsacales | Adoxaceae | *Viburnum* | 224 |
| 96 | *Berchemiella wilsonii* | Rosales | Rhamnaceae | *Berchemiella* | 216 |
| 97 | *Eragrostis* sp. | Poales | Poaceae | *Eragrostis* | 208 |
| 98 | *Macleaya cordata* | Ranunculales | Papaveraceae | *Macleaya* | 206 |
| 99 | *Viburnum erosum* | Dipsacales | Adoxaceae | *Viburnum* | 204 |
| 100 | *Smilax nipponica* | Liliales | Smilacaceae | *Smilax* | 202 |
